# Mucosal flap creation in colorectal endoscopic submucosal dissection using a V-shaped incision

**DOI:** 10.1055/a-2299-2407

**Published:** 2024-04-29

**Authors:** Koichi Hamada, Yoshinori Horikawa, Kae Techigawara, Takayuki Nagahashi, Masafumi Ishikawa, Michitaka Honda, Tamotsu Sugai

**Affiliations:** 1 13704 Department of Gastroenterology, Southern Tohoku Research Institute for Neuroscience Southern Tohoku General Hospital, Koriyama, Japan; 2 12775 Department of Minimally Invasive Surgical and Medical Oncology, Fukushima Medical University, Fukushima, Japan; 3 13704 Department of Surgery, Southern Tohoku Research Institute for Neuroscience Southern Tohoku General Hospital, Koriyama, Japan; 4 13704 Department of Pathology, Southern Tohoku Research Institute for Neuroscience Southern Tohoku General Hospital, Koriyama, Japan

The creation of a flap at the beginning of colorectal endoscopic submucosal dissection (ESD) of lesions on the semilunar fold or in the cecum is challenging because the knife is at right angles to the muscle layer. Thus, several innovations in flap creation have been reported 1 2 . The usefulness of traction-assisted ESD has been reported, including the clip-with-line 3 and the S-O clip 4 methods, which require a clip to be attached to the flap. Underwater ESD has also been reported to be helpful 5 ; however, it first requires the creation of a flap. The advantage of being able to create a flap safely and quickly, even in areas that are difficult to treat, is significant. Here, we report the successful creation of a mucosal flap using a V-shaped incision.

A 70-year-old man presented with a 38-mm type 0-IIa colonic adenocarcinoma of the cecum ( Fig. 1 ). ESD was performed using a DualKnife J (KD-655Q; Olympus, Tokyo, Japan) ( Video 1 ). The lesion was located where the knife encounters the muscle layer, and we anticipated that it would be challenging to create a flap and insert a hood between the mucosa and muscle layers. Furthermore, when the colon is dilated by insufflation, it is difficult for the endoscope to reach the lesion, and it is necessary to deflate in order to approach the lesion. After local injection of fluid into the submucosa, a V-shaped flap was created by means of a V-shaped incision ( Fig. 2 ). The sharp angle of the V was on the anorectal side, where it is easier to enter. The V-shaped incision created a narrow flap that allowed insertion of the hood under the mucosa after only two dissection attempts ( Fig. 3 ). Once the hood has been inserted, ESD can be performed safely using underwater or traction-assisted ESD, as appropriate.

**Fig. 1:**
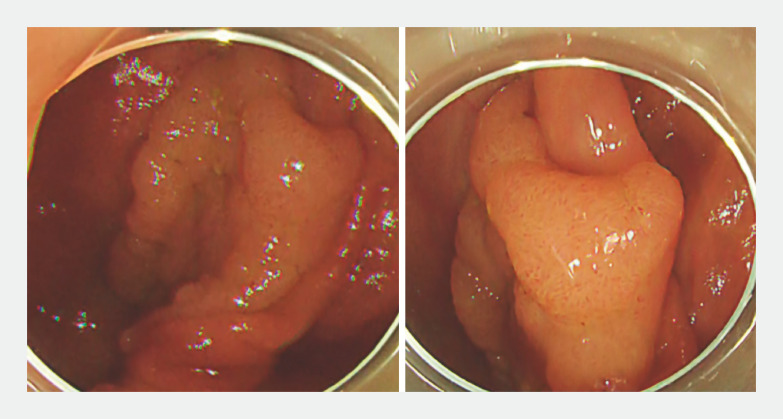
White light image of a 38-mm type 0-IIa colonic adenocarcinoma located on the fold of the cecum in a 70-year-old man.

**Fig. 2:**
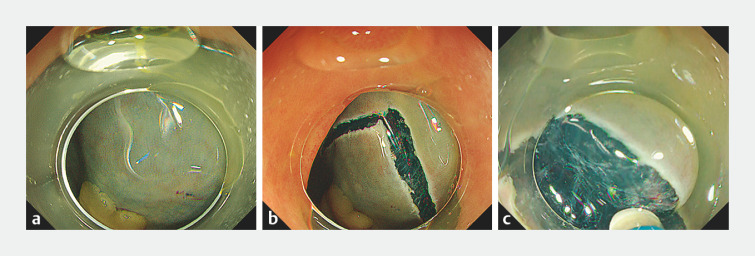
The V-shaped flap. **a** Determination of the apex of the V-shaped incision after submucosal injection. **b** Creation of the V-shaped incision. **c** A V-shaped incision has created a narrow flap, allowing the hood to be inserted under the mucosa after only two dissection attempts.

**Fig. 3:**
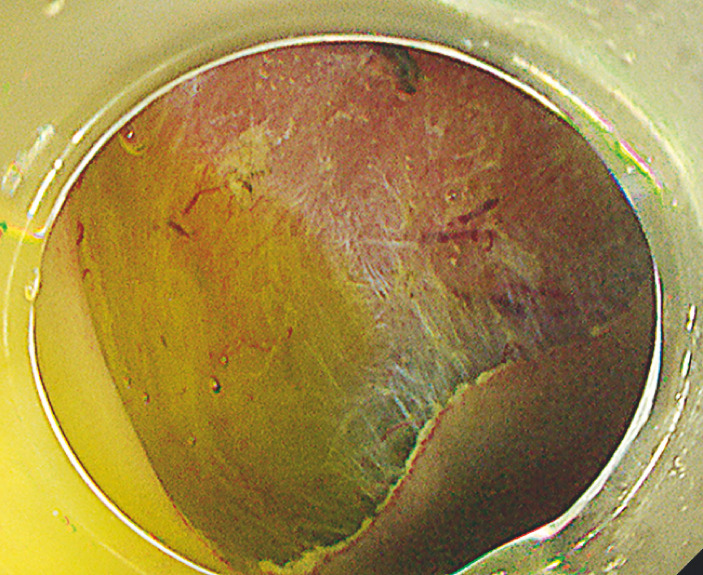
The ulcer without muscle damage observed after complete en bloc resection.

Mucosal flap creation using a V-shaped incision in colorectal endoscopic submucosal dissection.Video 1

In conclusion, we have demonstrated how a V-shaped incision can create a narrow flap for colorectal ESD.

Endoscopy_UCTN_Code_CPL_1AJ_2AD_3AD

## Notes

Endoscopy E-Videos https://eref.thieme.de/e-videos
*E-Videos* is an open access online section of the journal *Endoscopy* , reporting on interesting cases and new techniques in gastroenterological endoscopy. All papers include a high-quality video and are published with a Creative Commons CC-BY license. Endoscopy E-Videos qualify for HINARI discounts and waivers and eligibility is automatically checked during the submission process. We grant 100% waivers to articles whose corresponding authors are based in Group A countries and 50% waivers to those who are based in Group B countries as classified by Research4Life (see: https://www.research4life.org/access/eligibility/ ). This section has its own submission website at https://mc.manuscriptcentral.com/e-videos .
